# Supplementary figures and images for: Inotodiol induces hepatocellular carcinoma apoptosis by activation of MAPK/ERK pathway
Source: PLoS One. 2025 Jan 29;20(1):e0318450. doi: 10.1371/journal.pone.0318450 (PMC11778785; doi:10.1371/journal.pone.0318450)

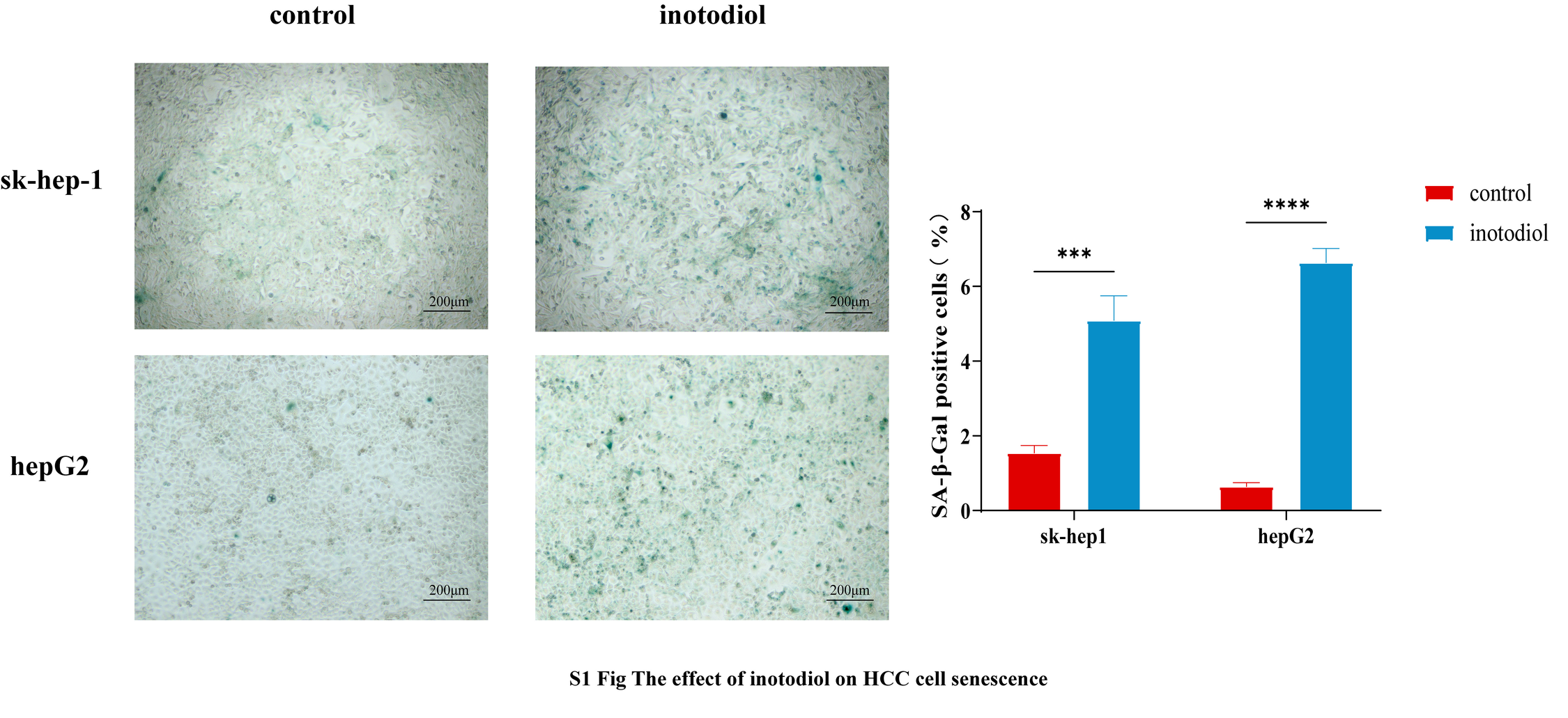

Supplement: S1 Fig — *significantly different from control group. ***p<0.001, ****p<0.0001, n = 3. (TIF) [file pone.0318450.s001.tif]

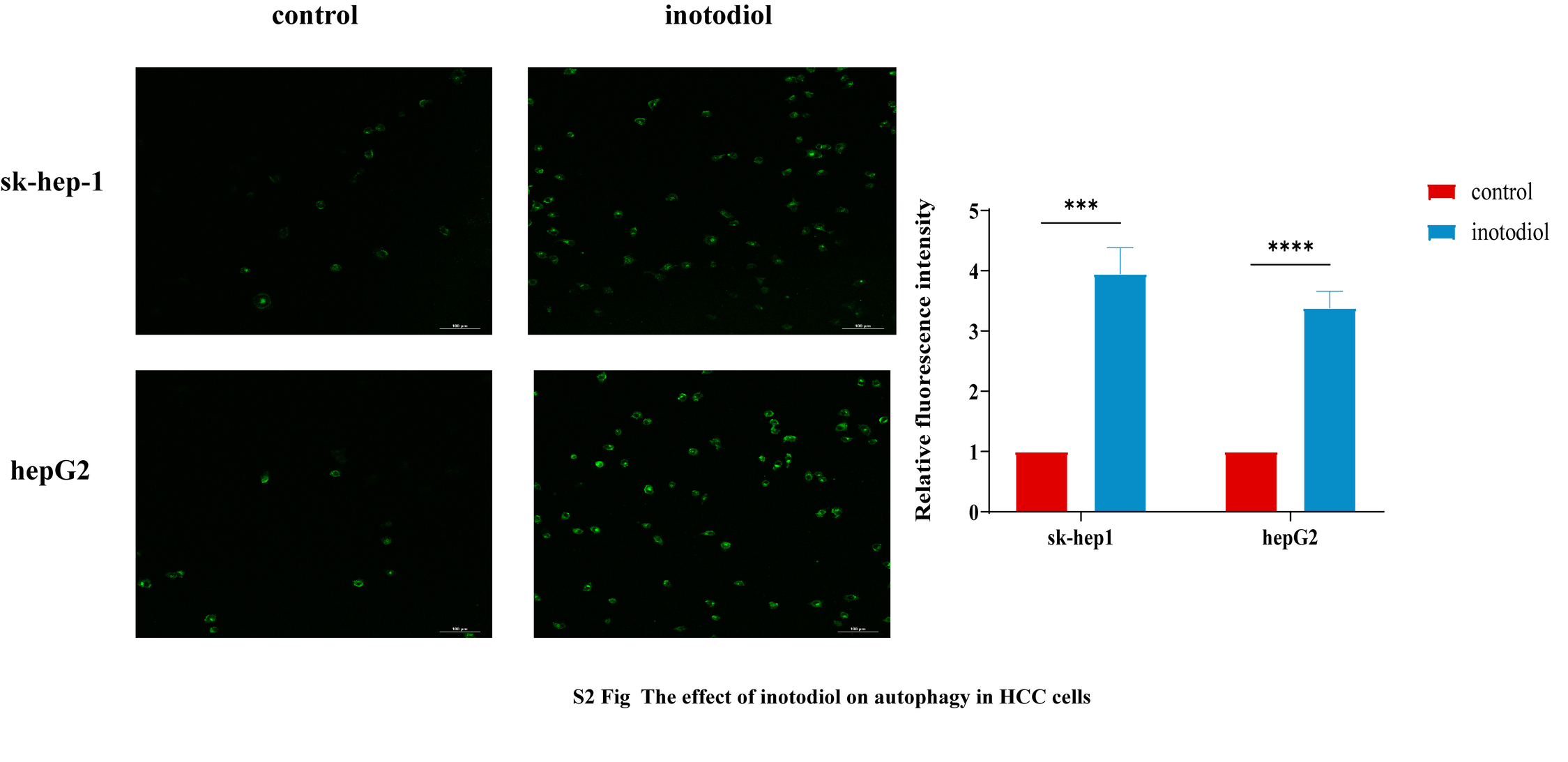

Supplement: S2 Fig — ***p<0.001, ****p<0.0001, n = 3. (TIF) [file pone.0318450.s002.tif]

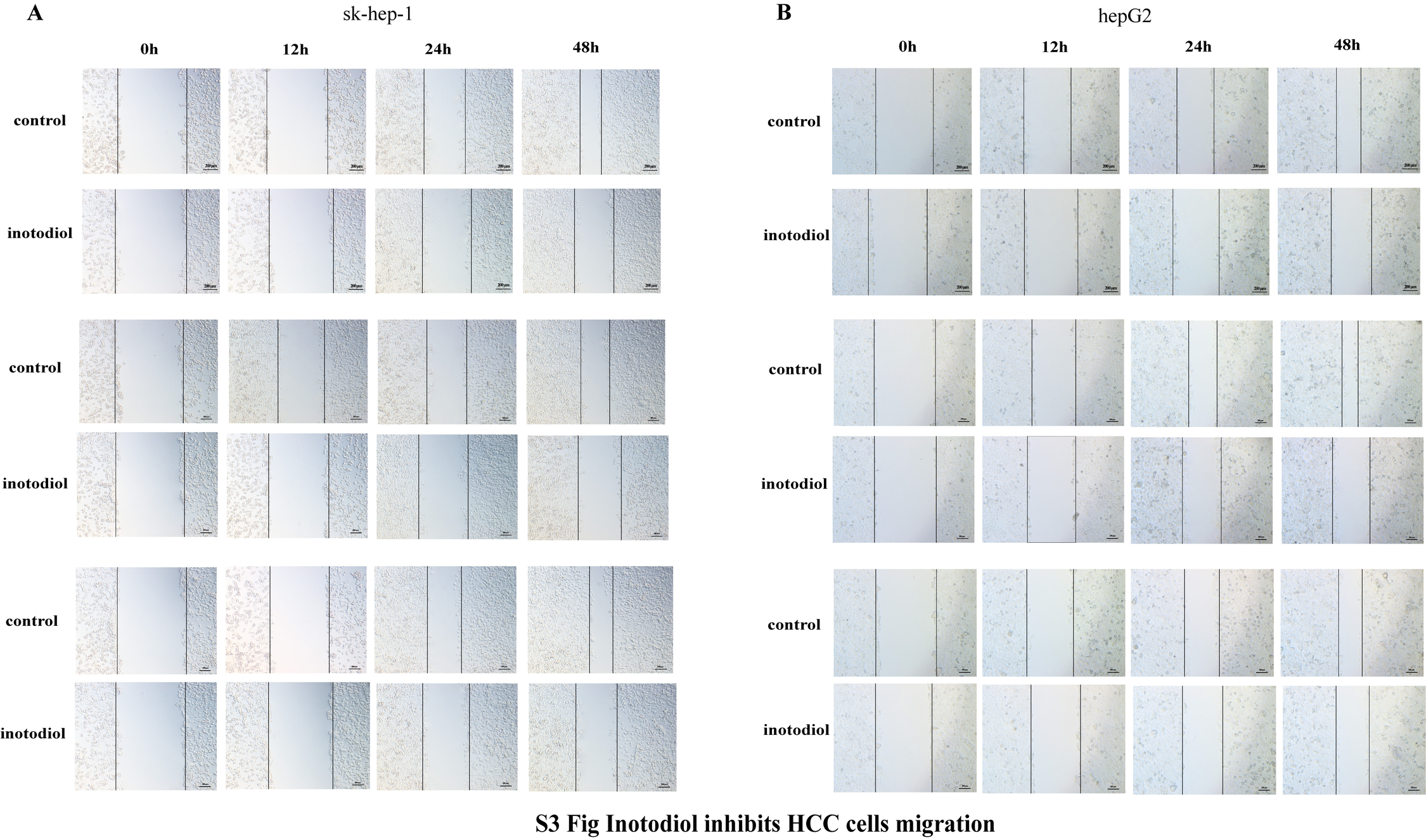

Supplement: S3 Fig — (TIF) [file pone.0318450.s003.tif]

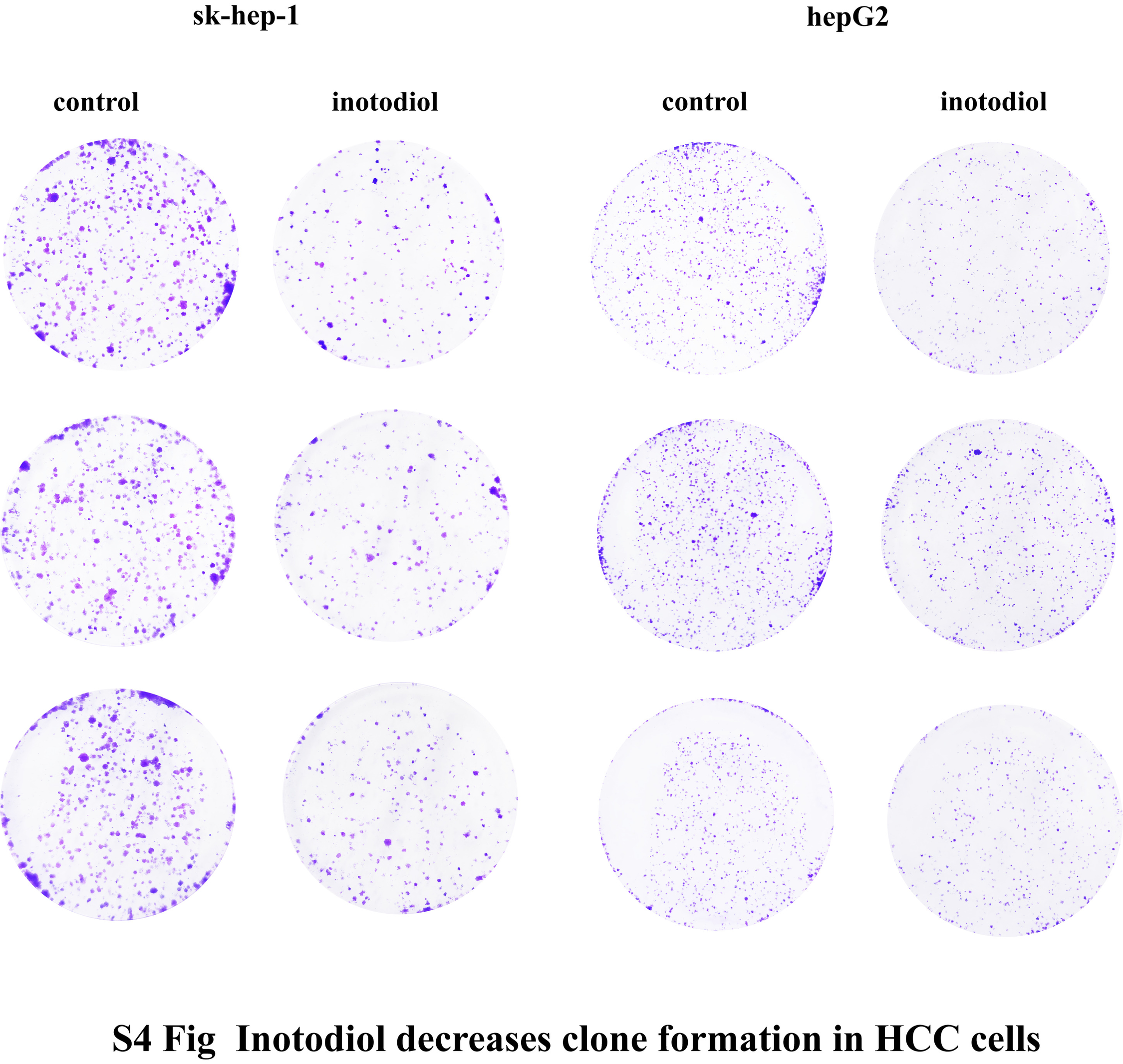

Supplement: S4 Fig — (TIF) [file pone.0318450.s004.tif]

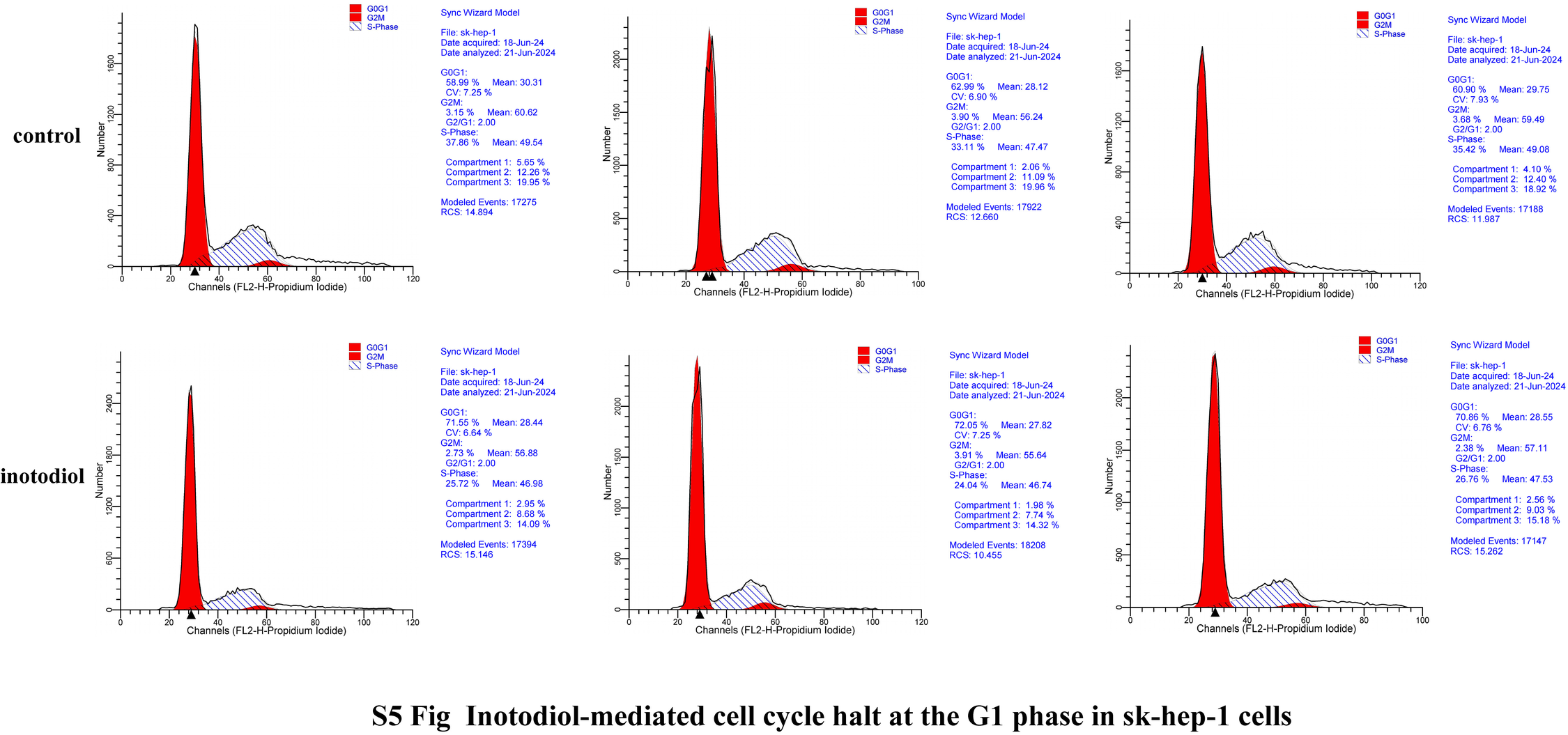

Supplement: S5 Fig — (TIF) [file pone.0318450.s005.tif]

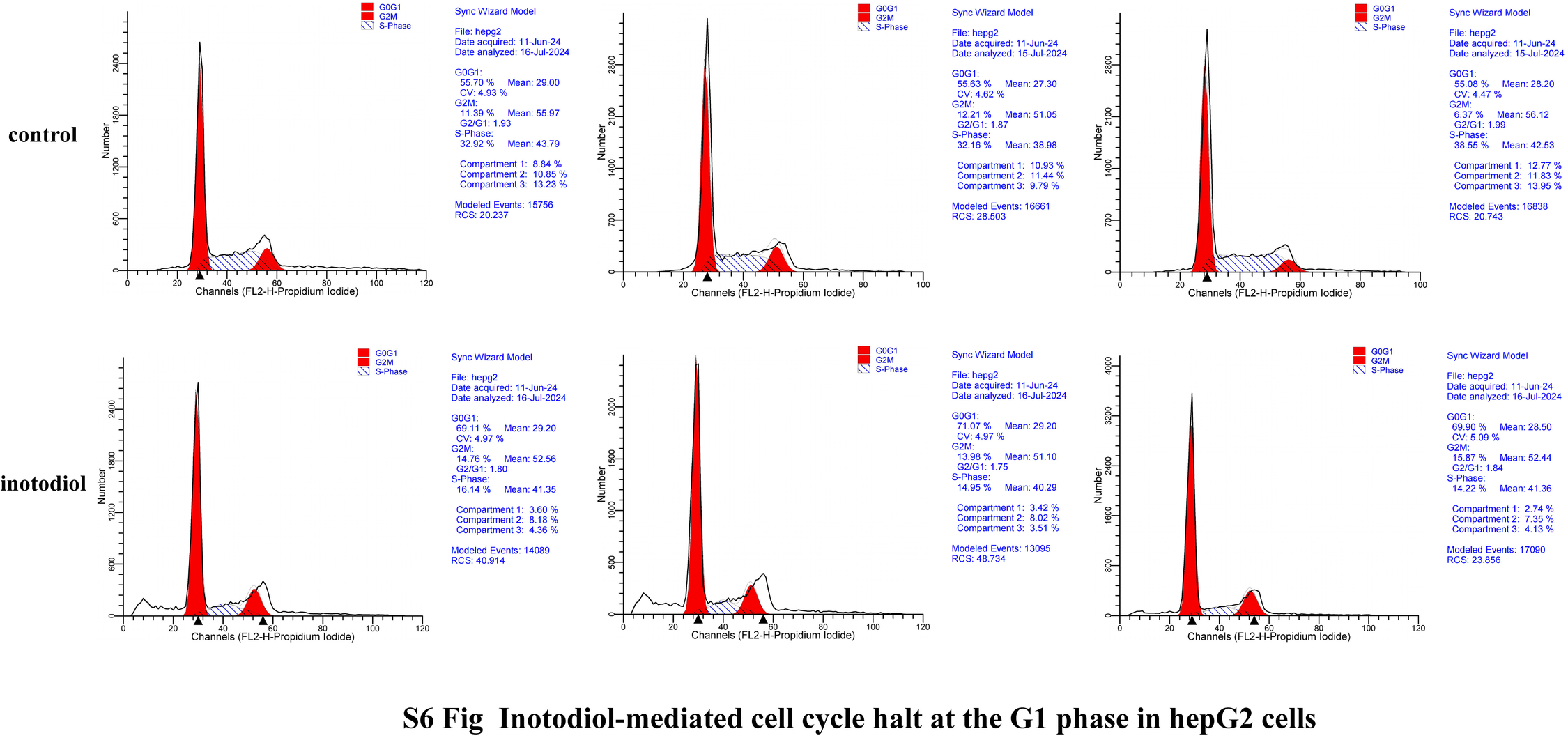

Supplement: S6 Fig — (TIF) [file pone.0318450.s006.tif]

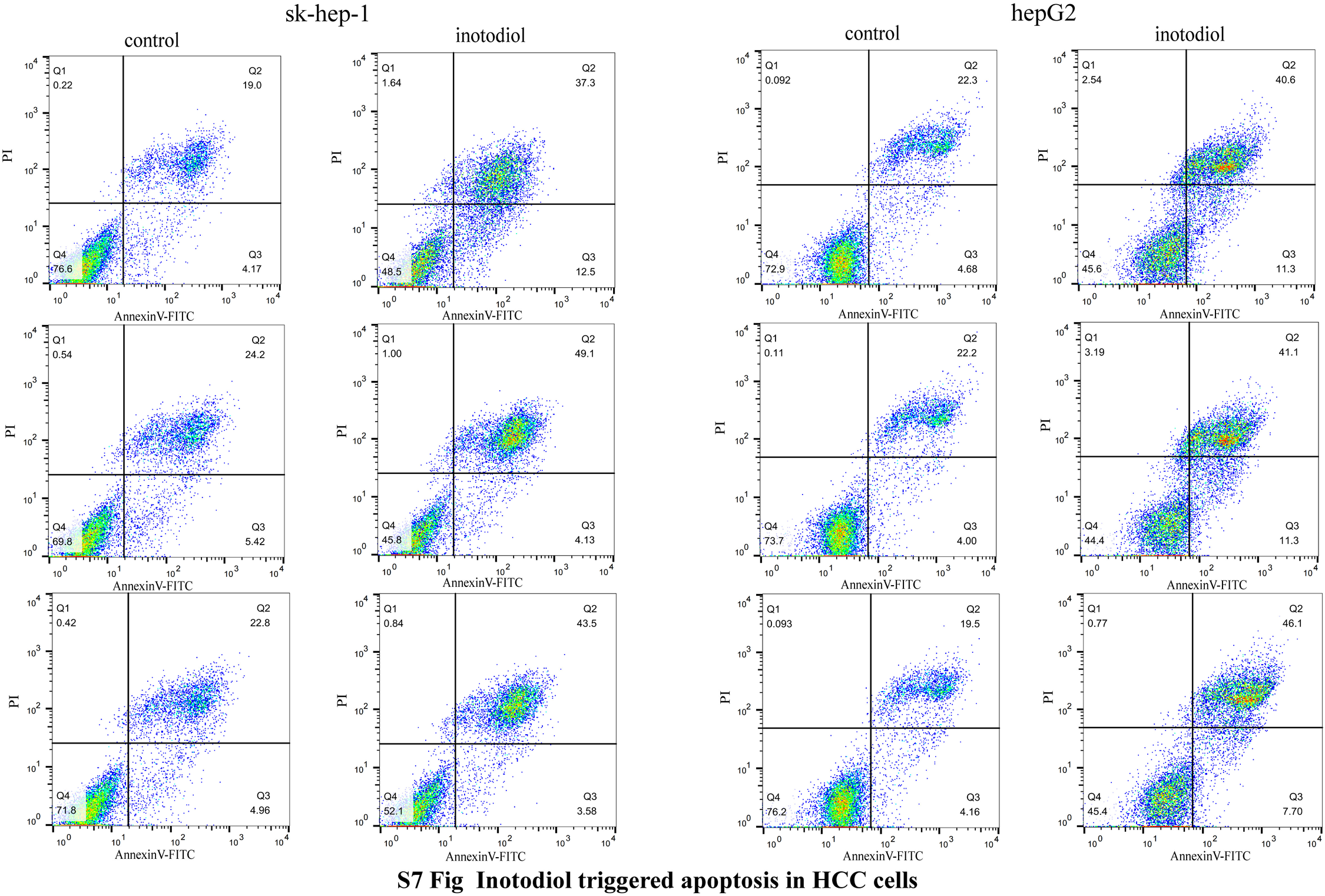

Supplement: S7 Fig — (TIF) [file pone.0318450.s007.tif]

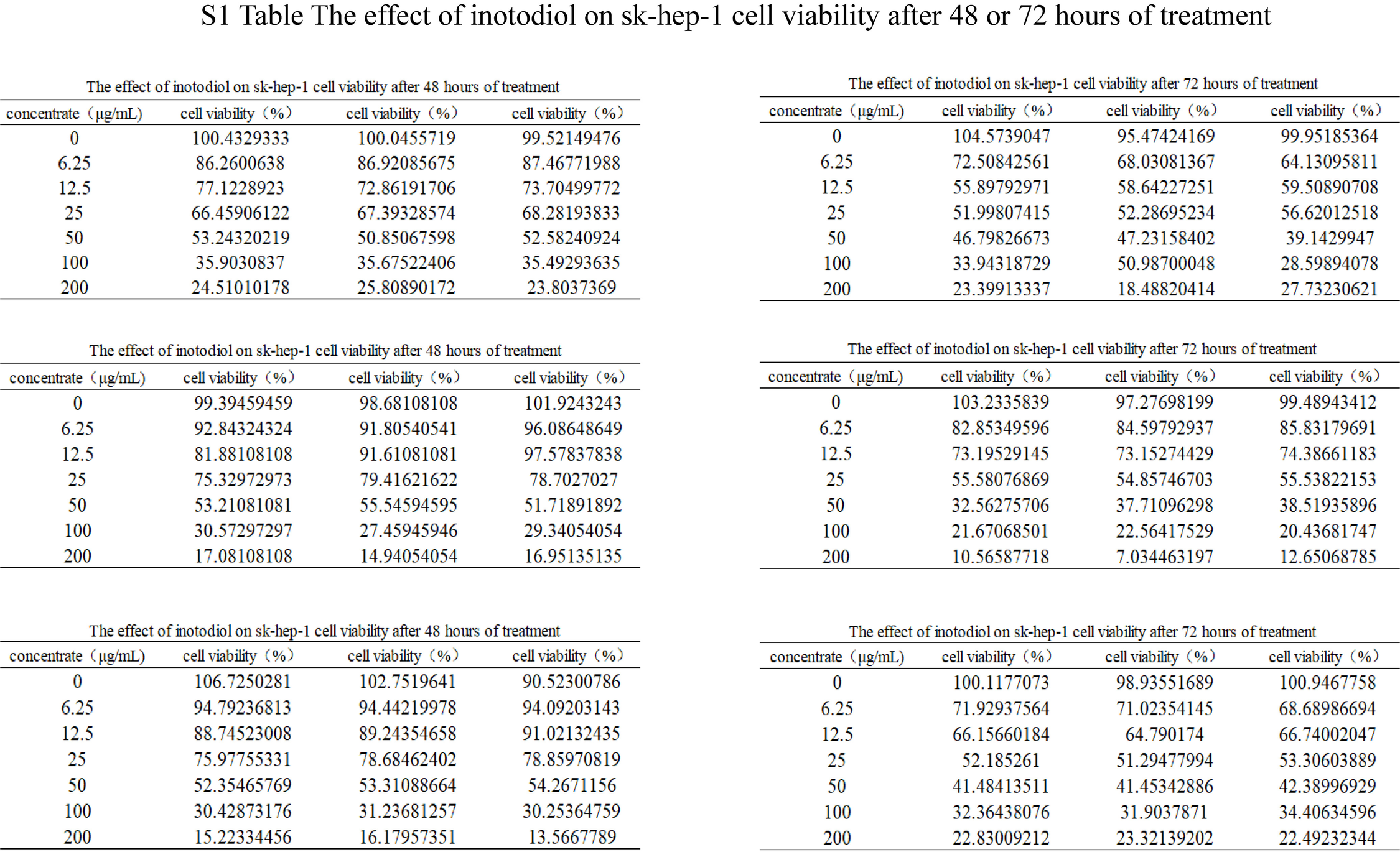

Supplement: S1 Table — (TIF) [file pone.0318450.s008.tif]

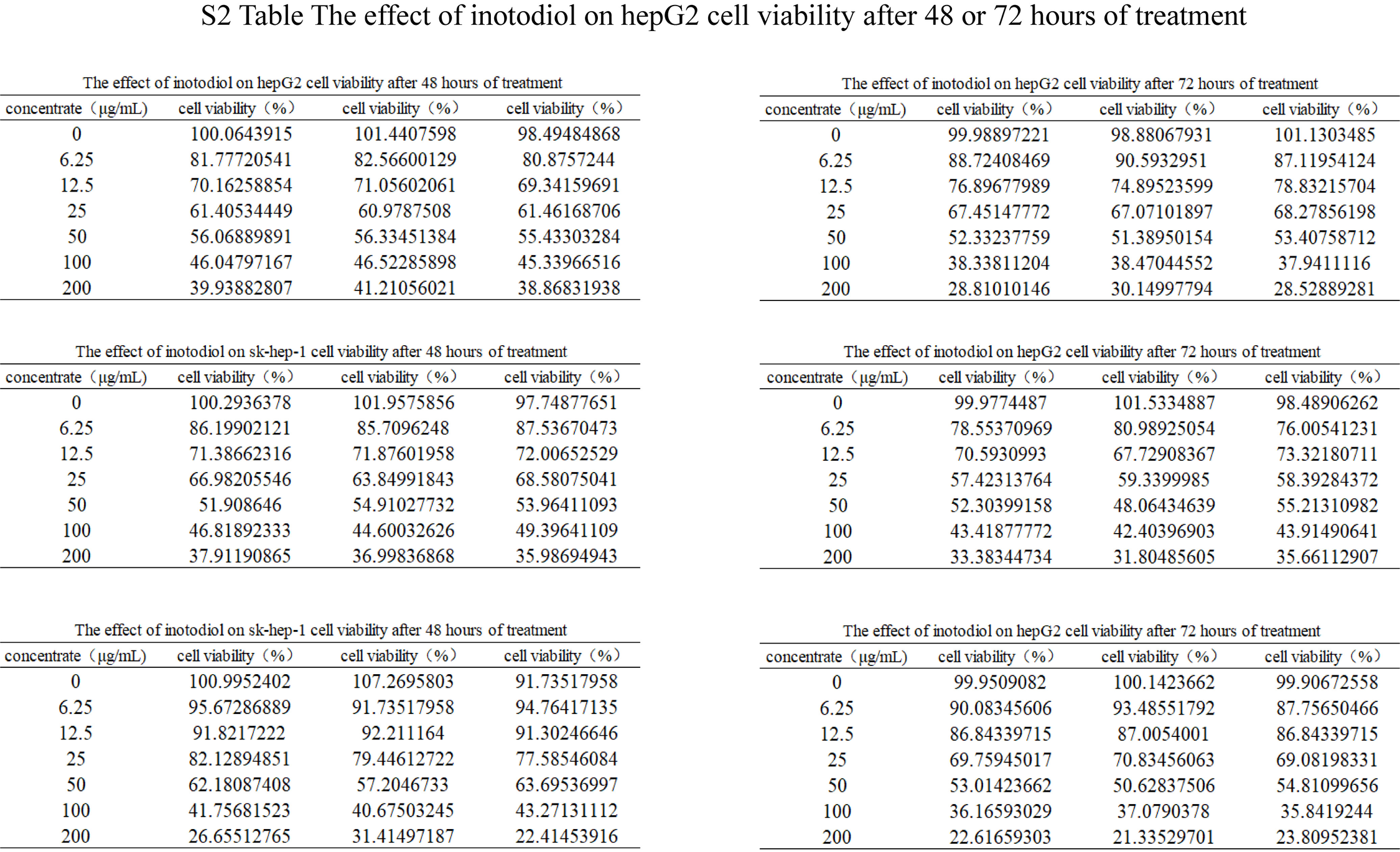

Supplement: S2 Table — (TIF) [file pone.0318450.s009.tif]

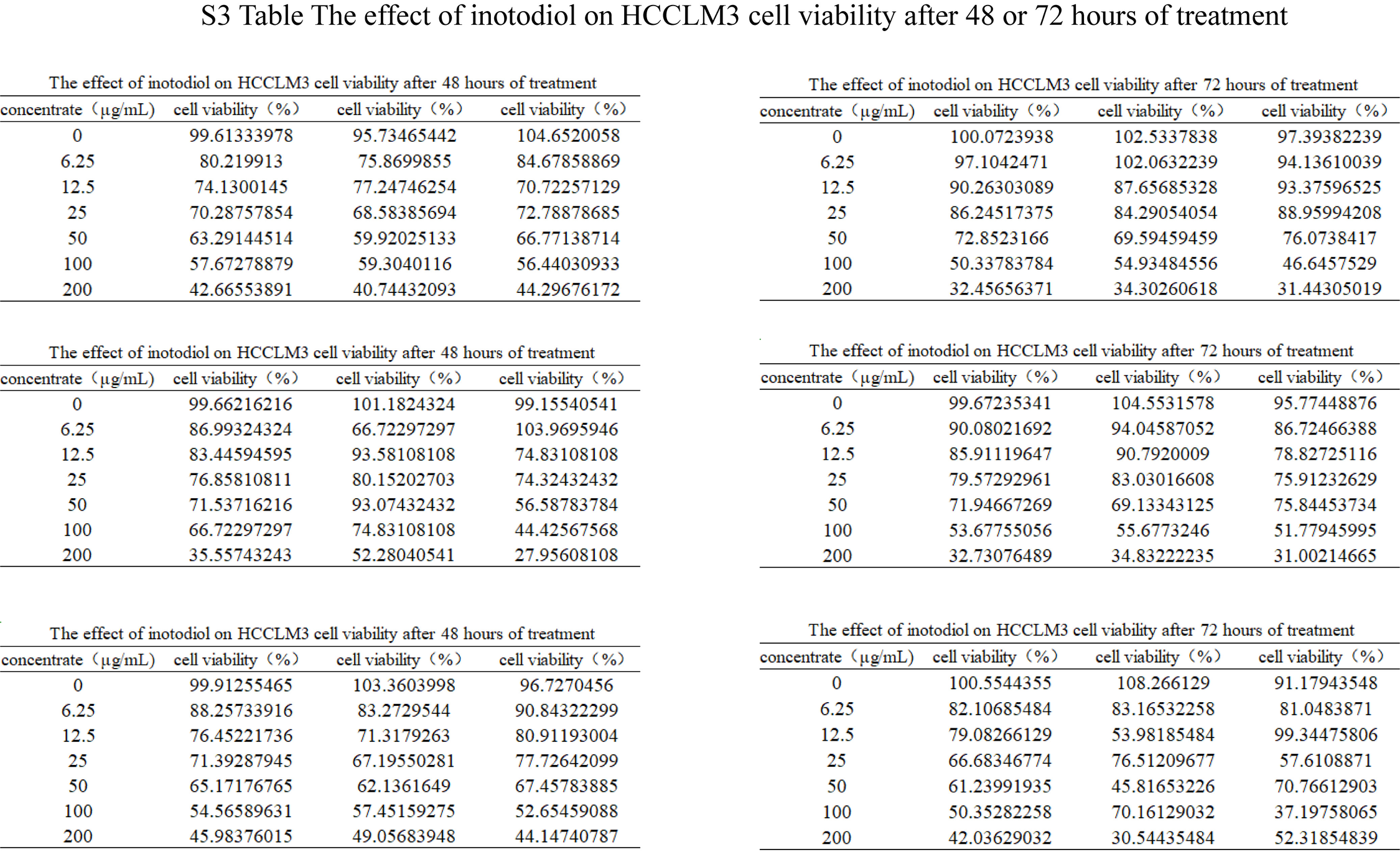

Supplement: S3 Table — (TIF) [file pone.0318450.s010.tif]

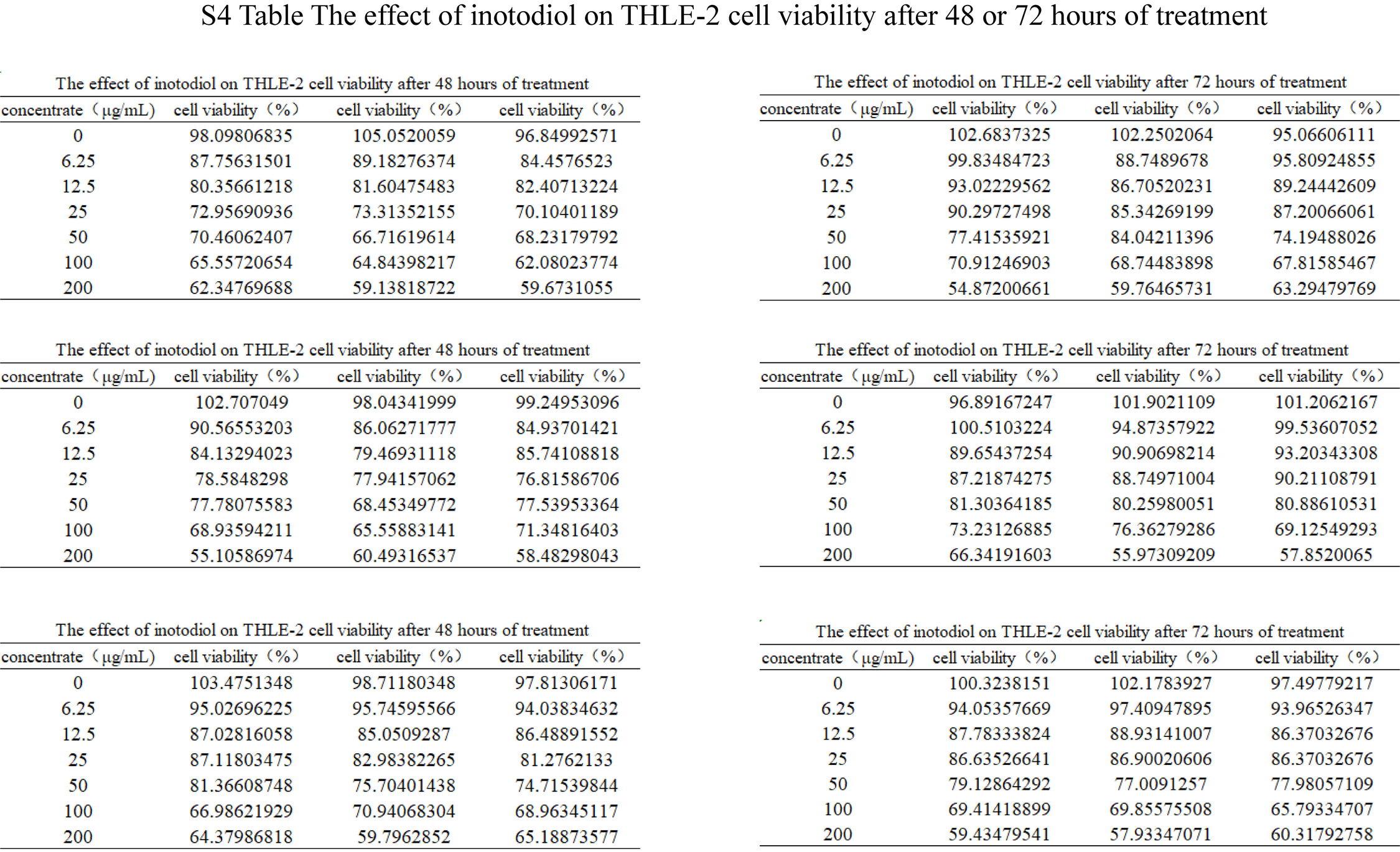

Supplement: S4 Table — (TIF) [file pone.0318450.s011.tif]

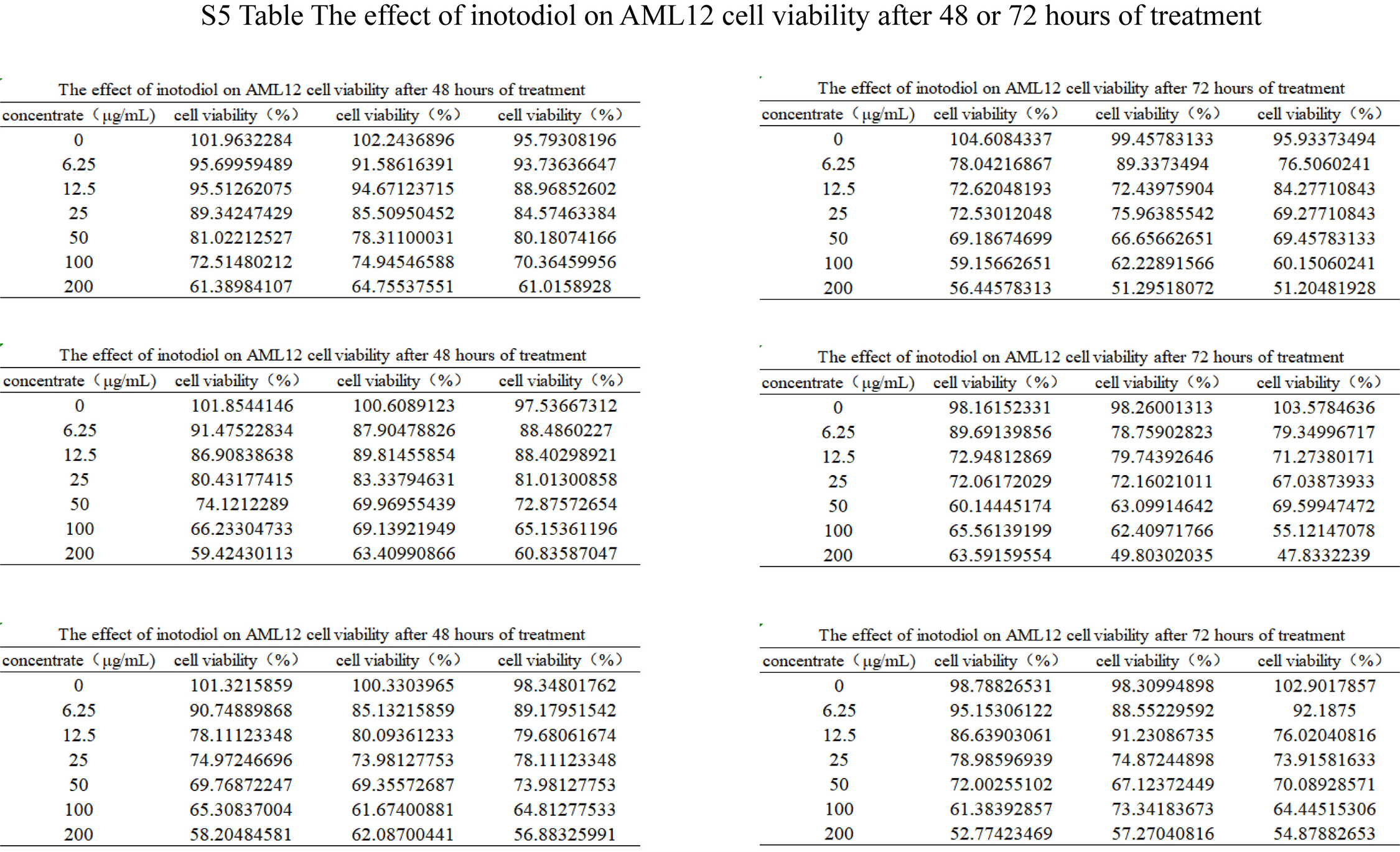

Supplement: S5 Table — (TIF) [file pone.0318450.s012.tif]

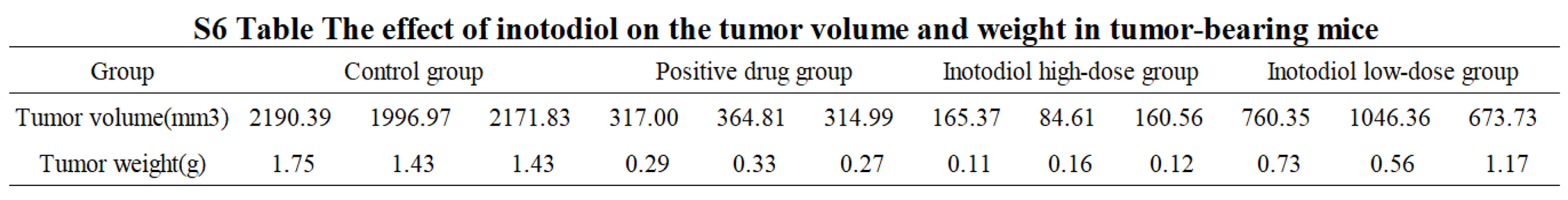

Supplement: S6 Table — (TIF) [file pone.0318450.s013.tif]

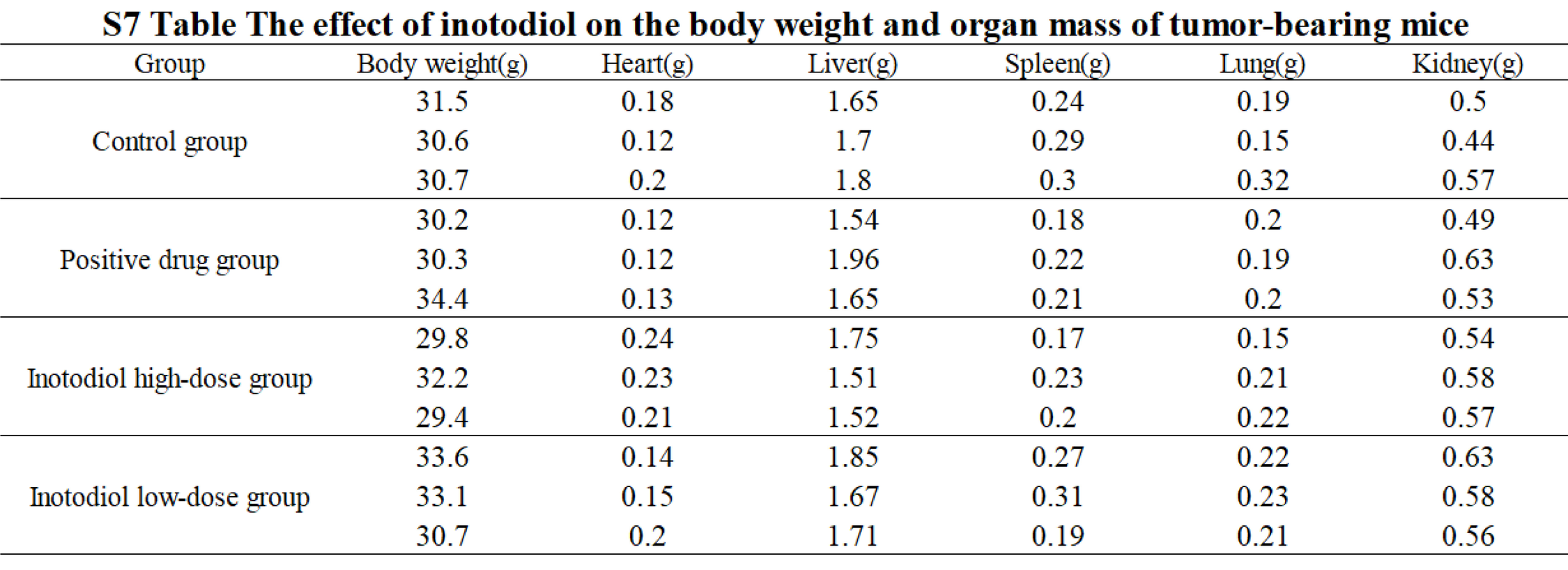

Supplement: S7 Table — (TIF) [file pone.0318450.s014.tif]
