# Supplementary material for: Inotodiol induces hepatocellular carcinoma apoptosis by activation of MAPK/ERK pathway
Source: PLoS One. 2025 Jan 29;20(1):e0318450. doi: 10.1371/journal.pone.0318450 (PMC11778785; doi:10.1371/journal.pone.0318450)
Supplement: S1 Raw images — (PDF) [file pone.0318450.s015.pdf]

sk-hep-1

Below are the experimental results of the periodic-related protein. The loading order is consistently control group 1, inotodiol group 1, control group 2, inotodiol group 2, control group 3, inotodiol group 3. The loading amount and order of the internal reference tubulin are the same as that of the target protein, and the western blot experiment was completed under the same conditions and at the same time.

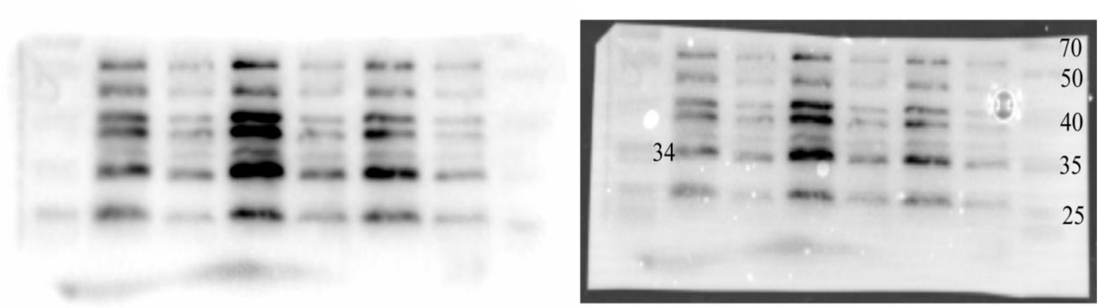

CDK2 34kDa

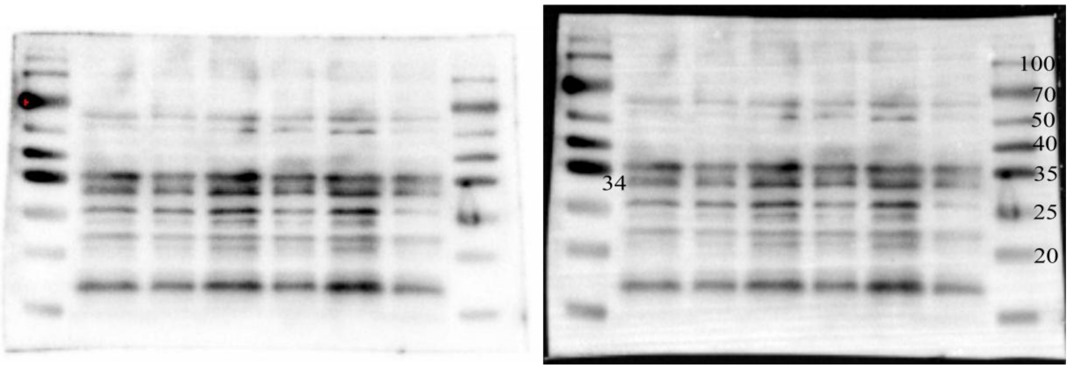

CDK4 34kDa

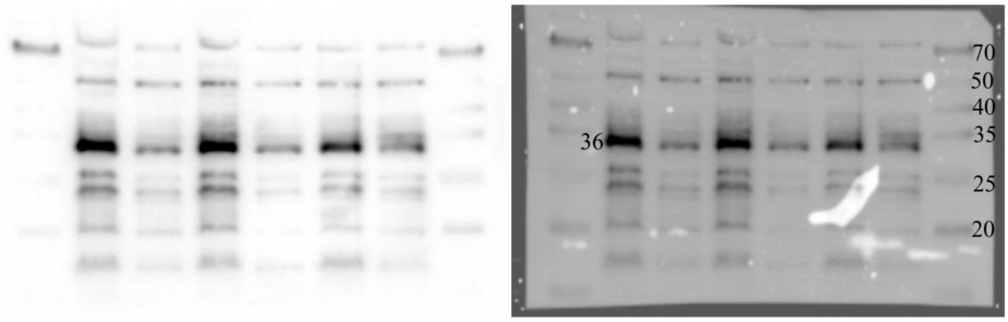

CDK6 36kDa

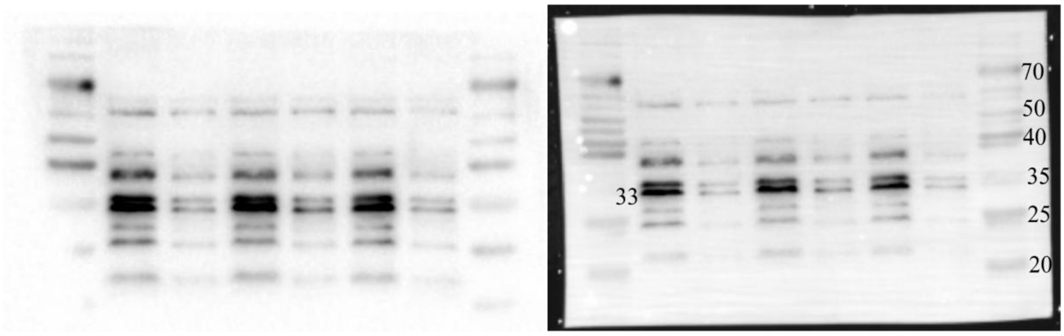

Cyclin D 33kDa

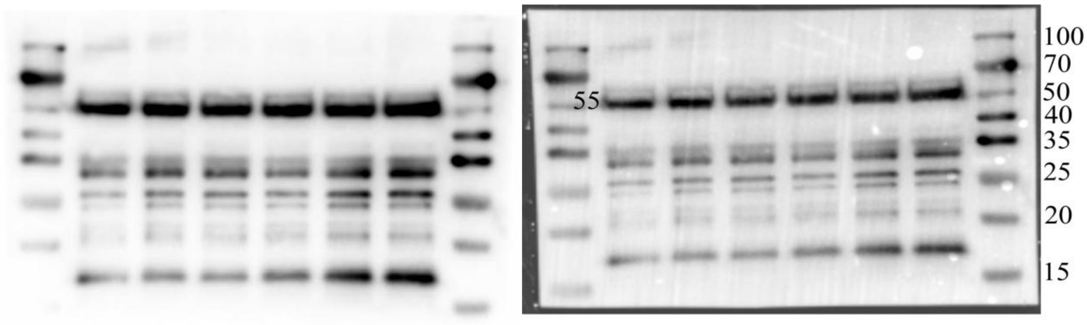

Tubulin 55kDa

Below are the experimental results of the apoptosis-related protein. The loading order is consistently control group 1, inotodiol group 1, control group 2, inotodiol group 2, control group 3, inotodiol group 3. The loading amount and order of the internal reference GAPDH are the same as that of the target protein, and the western blot experiment was completed under the same conditions and at the same time.

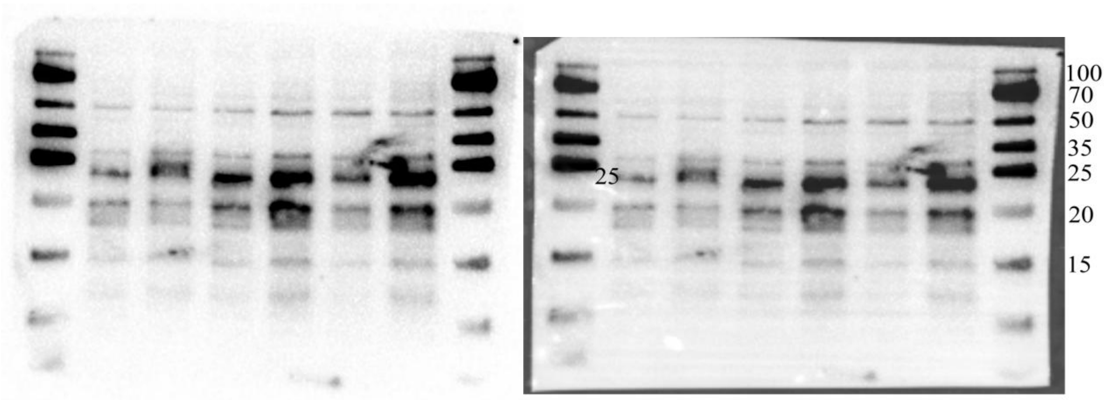

c-PARP1 25kDa

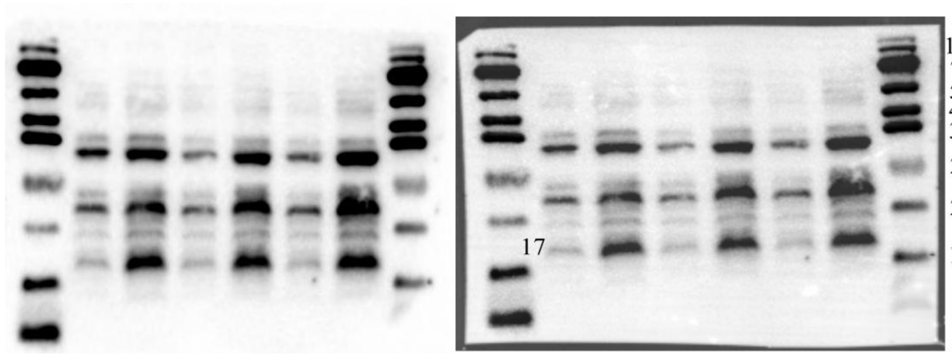

c-caspase3 17kDa

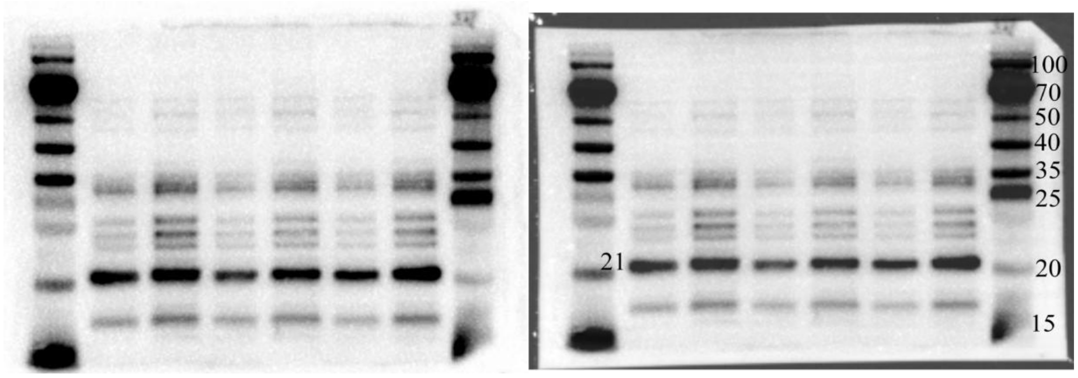

BAX 21kDa

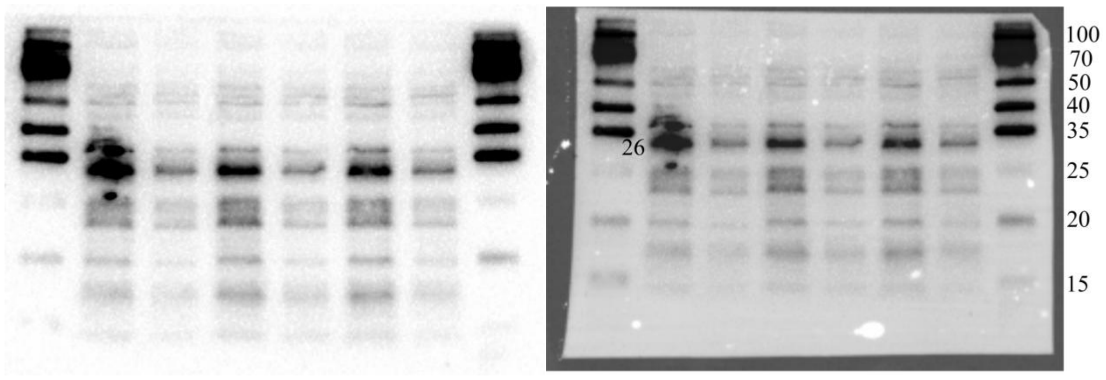

Bcl-2 26kDa

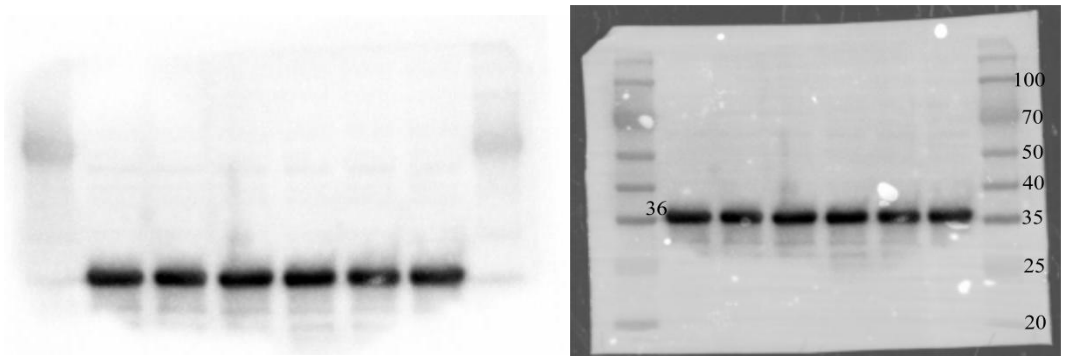

GAPDH 36kDa

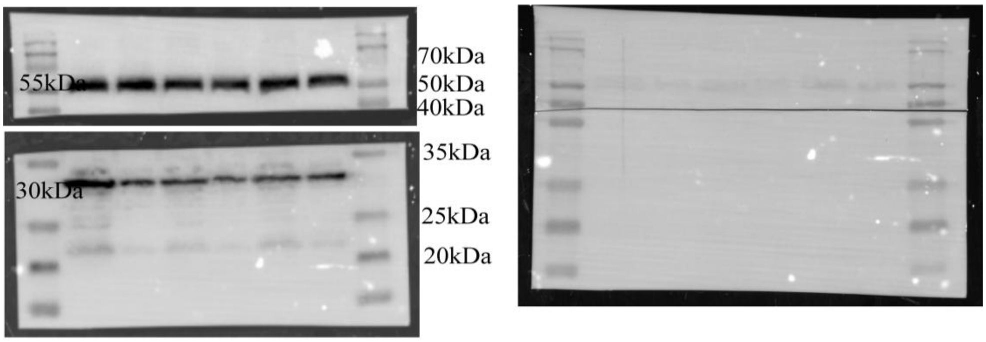

Tubulin 55kDa
Bcl-XL 30kDa

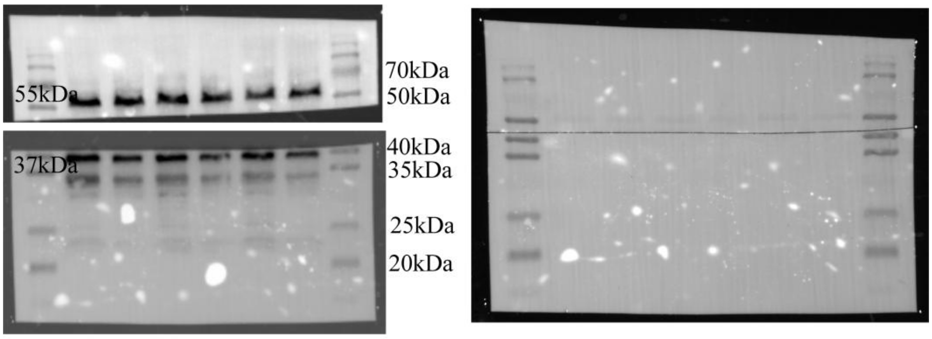

Tubulin 55kDa
MCL1 37kDa

Below are the experimental results of the proteins related to the MAPK/ERK pathway. The loading order is consistently control group 1, inotodiol group 1, control group 2, inotodiol group 2, control group 3, inotodiol group 3. The loading amount and order of the internal reference GAPDH are the same as that of the target protein, and the western blot experiment was completed under the same conditions and at the same time.

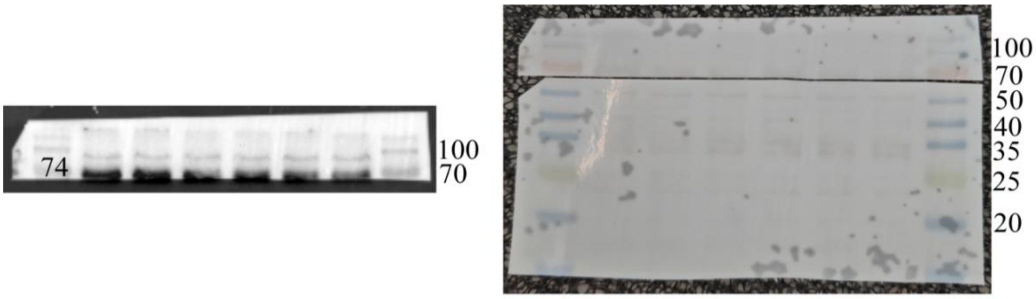

c-RAF 74kDa

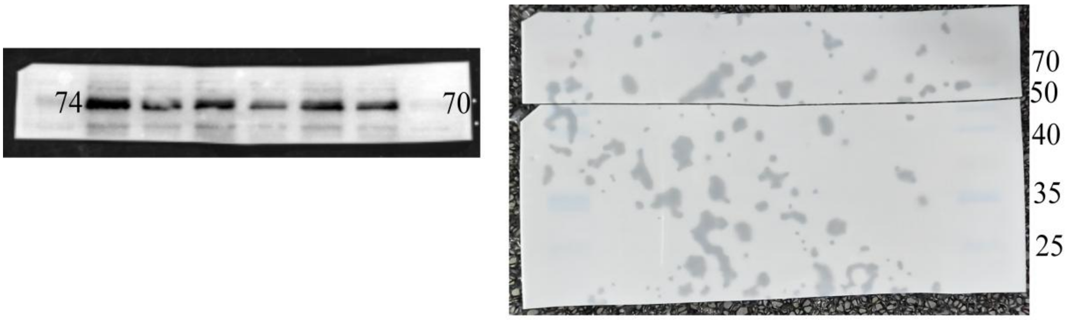

p-c-RAF 74kDa

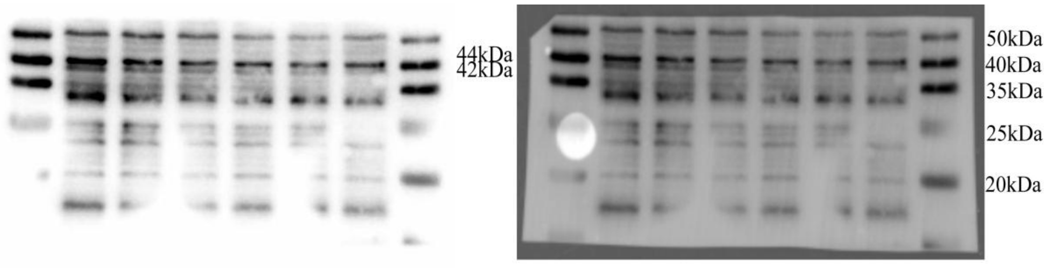

ERK1/2 42/44kDa

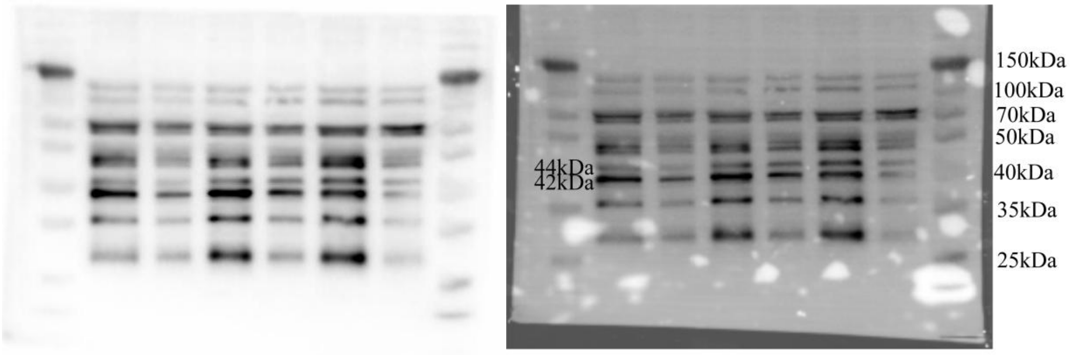

p-ERK1/2 42/44kDa

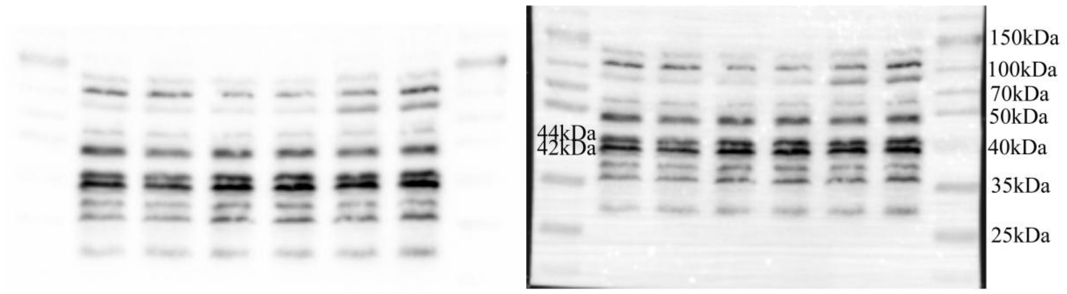

MEK1/2 42/44kDa

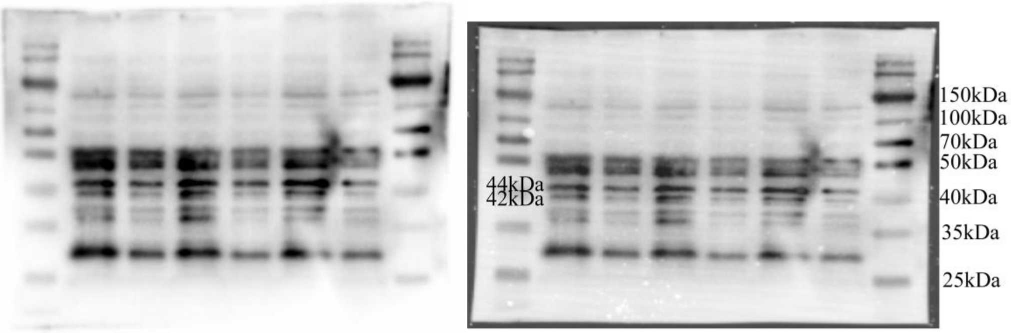

P-MEK1/2 42/44kDa

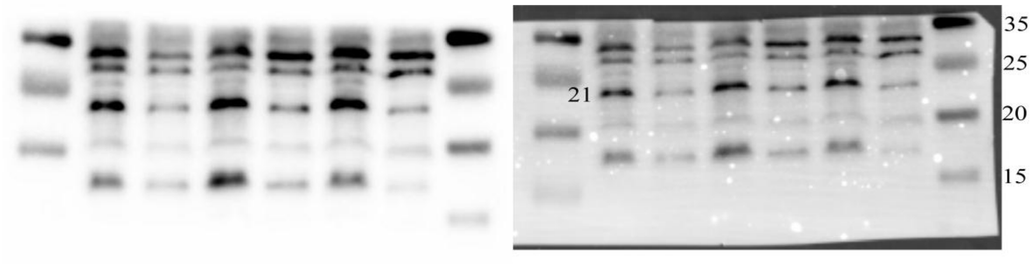

RAS 21kDa

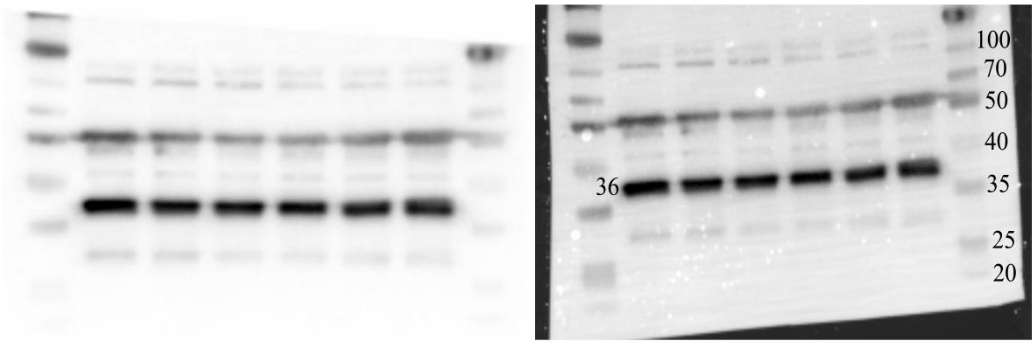

GAPDH 36kDa

HepG2

Below are the experimental results of the periodic-related protein. The loading order is consistently control group 1, inotodiol group 1, control group 2, inotodiol group 2, control group 3, inotodiol group 3. The loading amount and order of the internal reference tubulin are the same as that of the target protein, and the western blot experiment was completed under the same conditions and at the same time.

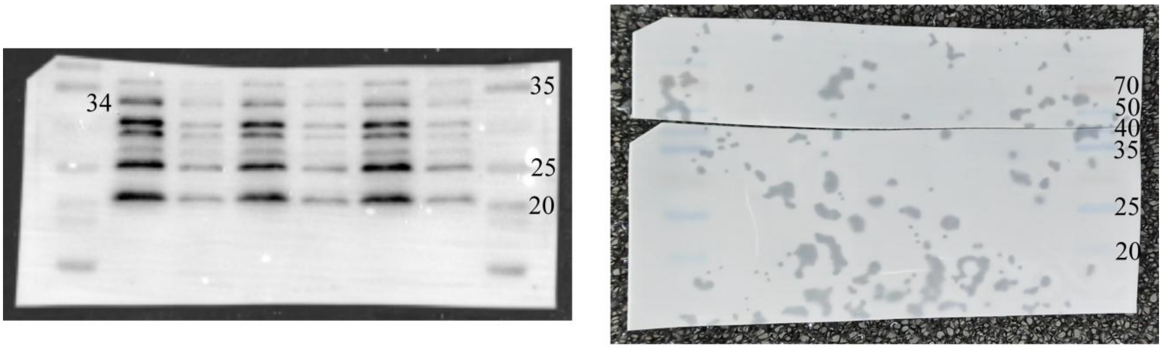

CDK2 34kDa

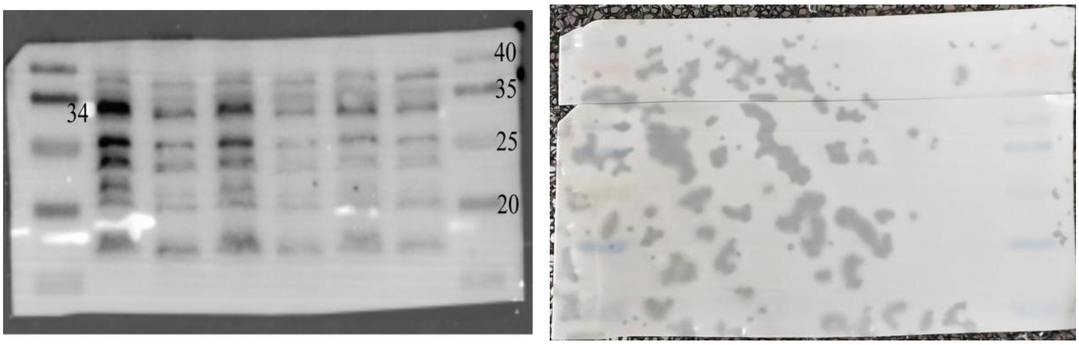

CDK4 34kDa

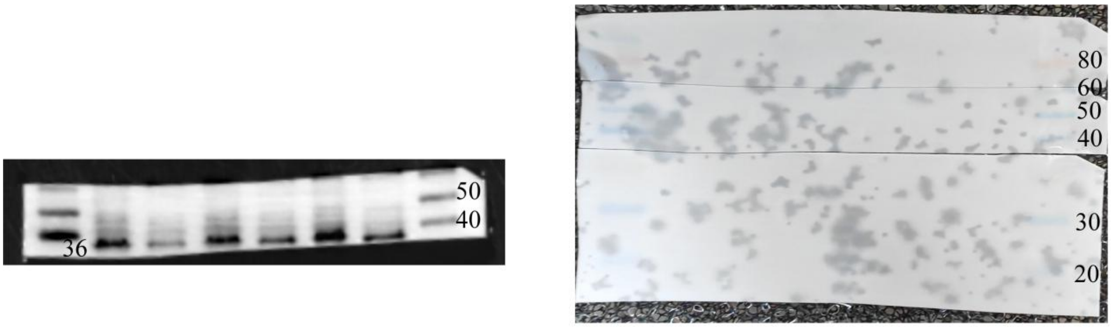

CDK6 36kDa

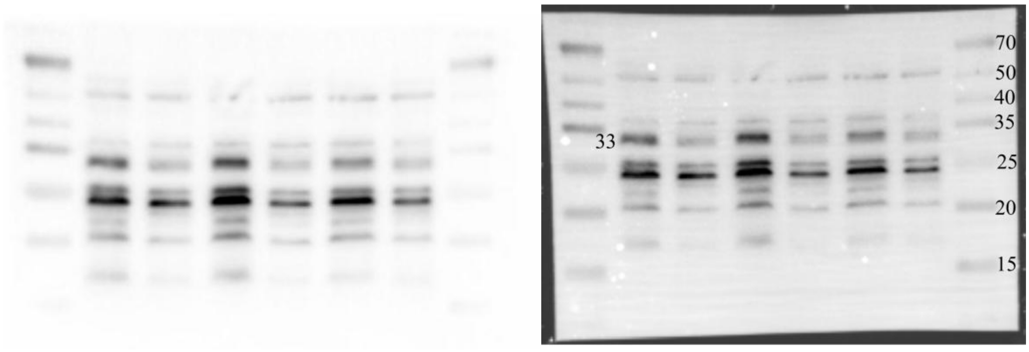

Cyclin D 33kDa

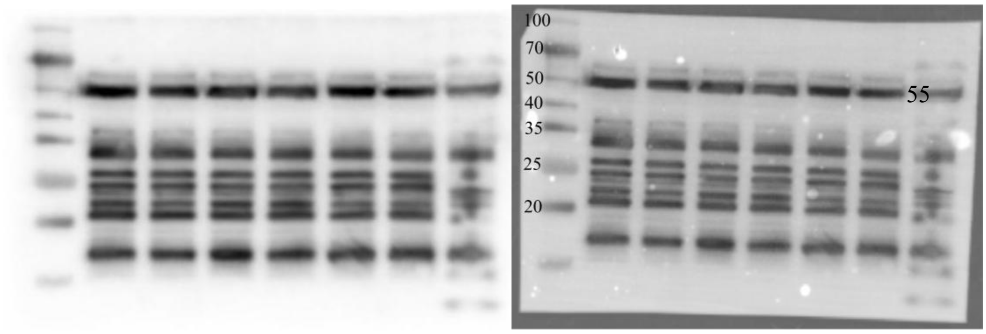

Tubulin 55kDa

Below are the experimental results of the apoptosis-related protein. The loading order is consistently control group 1, inotodiol group 1, control group 2, inotodiol group 2, control group 3, inotodiol group 3. The loading amount and order of the internal reference GAPDH are the same as that of the target protein, and the western blot experiment was completed under the same conditions and at the same time.

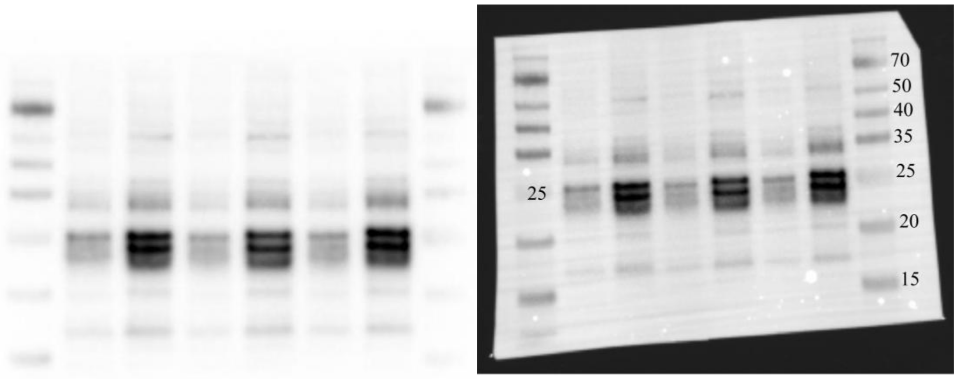

c-PARP1 25kDa

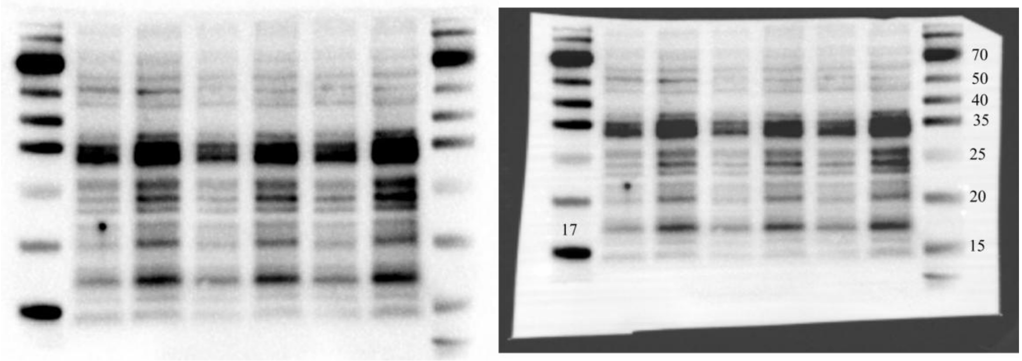

c-caspase3 17kDa

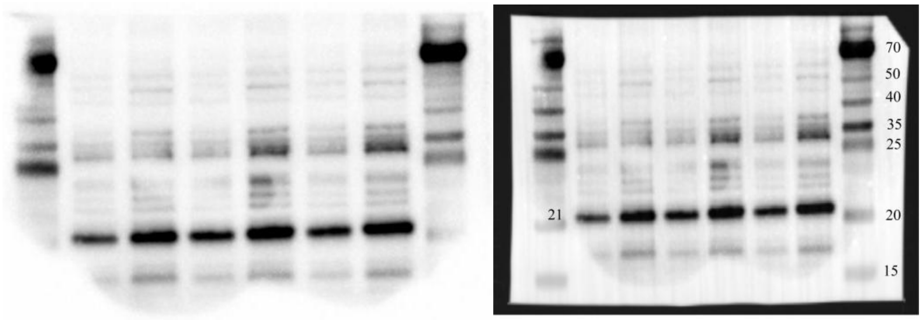

BAX 21kDa

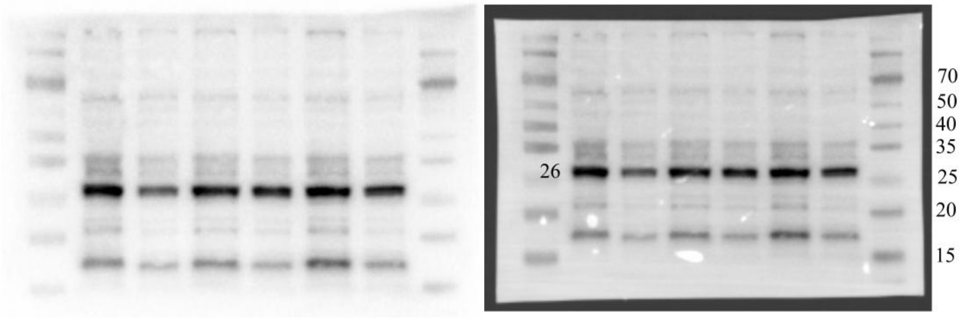

bcl-2 26kDa

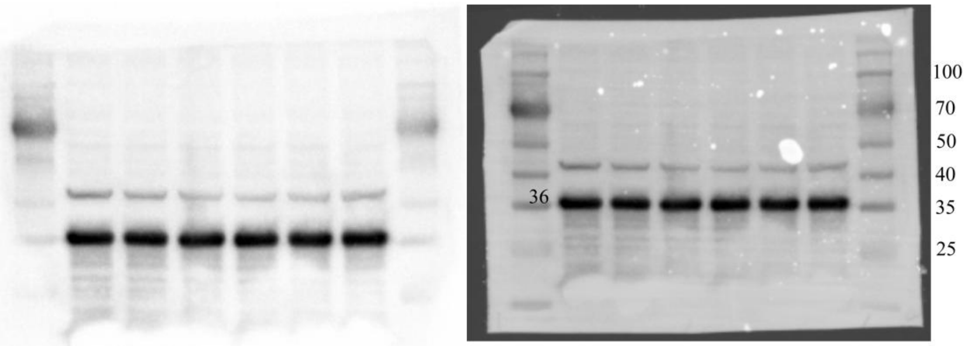

GAPDH 36kDa

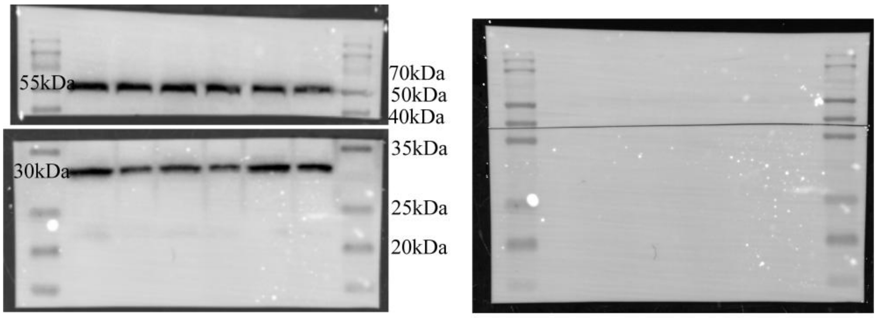

Tubulin 55kDa
Bcl-XL 30kDa

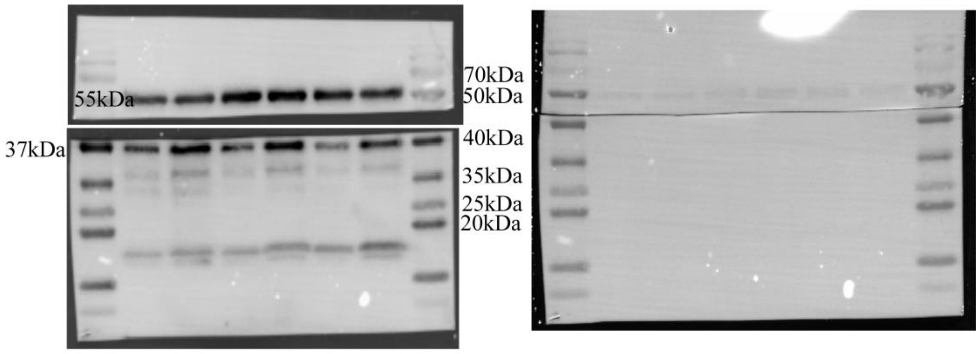

Tubulin 55kDa
MCL1 37kDa

Below are the experimental results of the proteins related to the MAPK/ERK pathway. The loading order is consistently control group 1, inotodiol group 1, control group 2, inotodiol group 2, control group 3, inotodiol group 3. The loading amount and order of the internal reference GAPDH are the same as that of the target protein, and the western blot experiment was completed under the same conditions and at the same time.

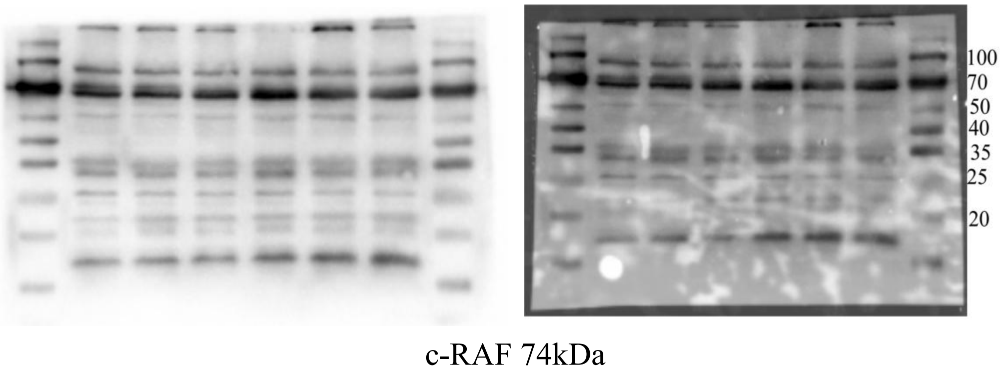

c-RAF 74kDa

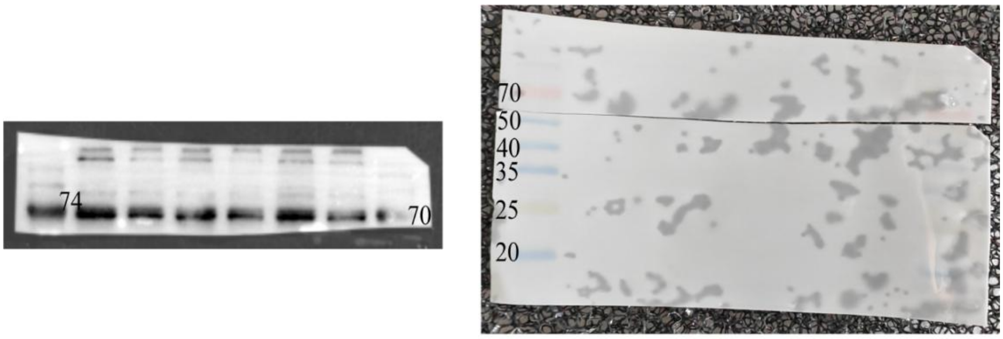

p-c-RAF 74kDa

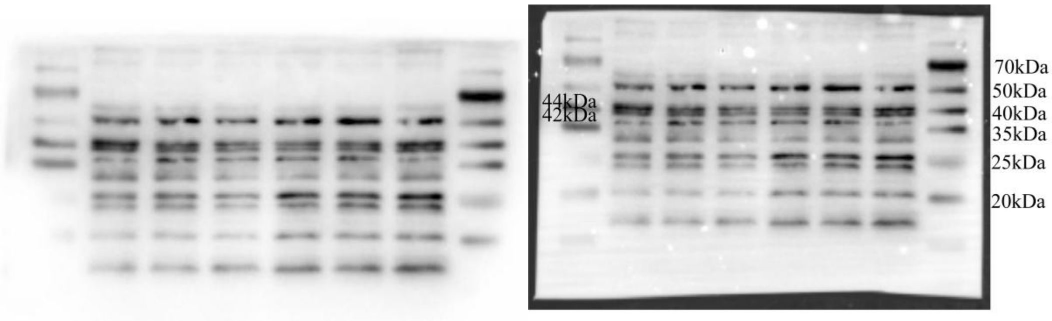

ERK1/2 42/44kDa

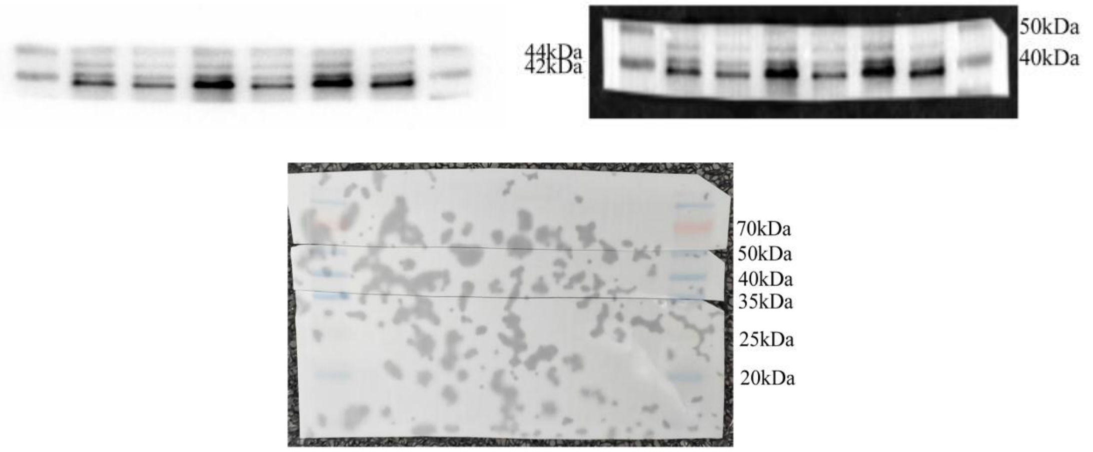

p-ERK1/2 42/44kDa

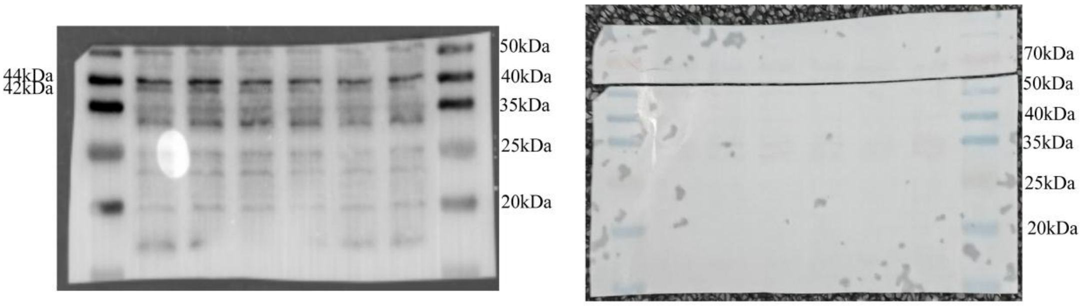

MEK1/2 42/44kDa

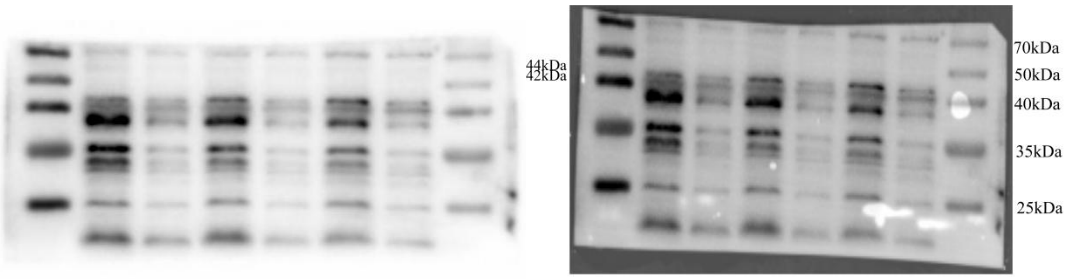

p-MEK1/2 42/44kDa

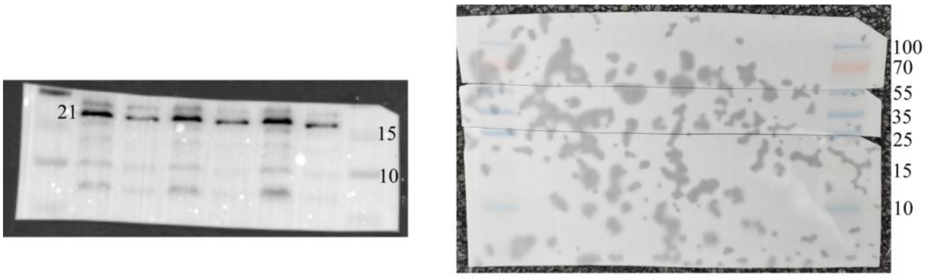

RAS 21kDa

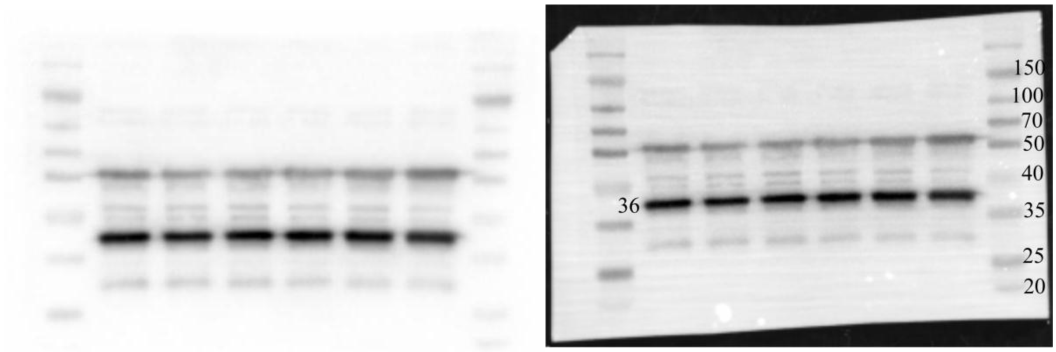

GAPDH 36kDa
