# Supplementary material for: Inotodiol induces hepatocellular carcinoma apoptosis by activation of MAPK/ERK pathway
Source: PLoS One. 2025 Jan 29;20(1):e0318450. doi: 10.1371/journal.pone.0318450 (PMC11778785; doi:10.1371/journal.pone.0318450)
Supplement: Attachment — Submitted filename: Response to Reviewers.docx [file pone.0318450.s016.docx]

Nov 23, 2024

Dear Dr. Wagdy M. Eldehna,

Thank you very much for your e-mail dated 11-Oct-2024.

Now I am sending our revised manuscript entitled “Inotodiol induces hepatocellular carcinoma apoptosis by activation of MAPK/ERK pathway” (Manuscript number: PONE-D-24-40336) for which we received your e-mail and reviewers’ comments 11-Oct-2024.

We have carefully revised our manuscript according the reviewer's comments. Please find a revised manuscript marked in **red** showing major changes and the response to the reviewer's comments.

We hope this revised manuscript is acceptable.

With best regards,

Yours Sincerely

Yushuang Xing，

*Heilongjiang University of Traditional Chinese Medicine*

*Harbin 150040, China.*

*e-mail: 342937606@qq.com*

=====================================

**The responses to the Reviewer's comments:**

Reviewer 1:
Comments to the Author

The study provide nice insights for using the natural products in tumor suppression. The manuscript can be improved more. Here are some points：

Response: Thank you very much for your positive comments.

1. The authors reported the effect on BCL2, how about MCL1 and BCLxl
   Response: We have carefully reviewed your valuable suggestions regarding the supplementation of experimental data. Following your advice, we have conducted additional experiments to investigate the expression of Bcl-XL and MCL1 in this study. We employed Western blot to detect the expression levels of Bcl-XL and MCL1 proteins, with the specific results presented in Fig 5C and Fig 5D.
2. (critical) the authors showed show the effect of the drug on non-cancerous cells or fibroblasts relevant to hepatocytes

Response: In accordance with the suggestions you previously offered, we have supplemented the CCK8 experimental results regarding the effects of inotodiol on human liver cell line THLE-2 and mouse liver cell line AML12, with the specific results shown in Fig 1A-B.

3. I was wondering if the drug induce an autophagic or senescence response?

Response: Regarding senescence and autophagy, these are also issues that we are very interested in. We have supplemented experiments on the impact of inotodiol on the senescence and autophagy phenotypes of HepG2 and sk-hep-1 cells. The results of these experiments have been organized and presented in the supplementary data, presented in Fig S1 and Fig S2.

4. What is meaning of media contain 1640?
Response: These section have been revised in the manuscript based on your excellent suggestions.

5. The paper need more organization and collecting the subfigures into a main panel for each section, not scattered figures.

Response: Thank you for your feedback, we have adjusted the figures and presented them in a single integrated figure for the same panel.

6. Are the authors able to perform an in vivo/3D culture study?

Response: In response to your suggestions, we have supplemented our study with in vivo experiments to further validate the inhibitory activity of inotodiol against liver cancer. The specific experimental methods have been added to the manuscript and marked in red for easy identification. The results of the supplementary experiments are detailed in Fig 7A-B and Table 1.

Reviewer 2:

Comments to the Author

I have gone through the submitted manuscript titled, Inotodiol induces hepatocellular carcinoma apoptosis by activation of MAPK/ERK pathway. This study emphasizes the antitumor effect of Inotodiol against hepatocellular carcinoma illustrating its possible mechanistic effect. The manuscript is well-written and the claims are supported by the data. This manuscript will benefit from some minor revisions.

Response: Thank you very much for your positive comments.

1. Little grammatical mistakes including spaces should be revised.

Response: Thanks for your suggestion. We tried our best to improve the manuscript and made some changes to the manuscript. These changes will not influence the content and framework of the paper. And here we did not list the changes but marked in red in the revised paper. We appreciate for Reviewers’ warm work earnestly and hope that the correction will meet with approval.

2. The abbreviation section should be added.

Response: In response to your valuable feedback, we have added a glossary of abbreviations at the end of the article to facilitate readers in consulting the professional terms and abbreviations used in the article more conveniently. This section lists in detail all the abbreviations and their full names that appear in the article, which helps to improve the readability and understandability of the article.We greatly appreciate your suggestion and believe that this improvement will make the article more complete.

3. Funding sources should be illustrated in the manuscript.

Response: Following your suggestion, we have added a section on funding sources in the manuscript to ensure transparency and comply with academic publishing standards. This section provides detailed information on the funding sources that supported this research, including the names of the funding agencies, project numbers, and their specific contributions to the study.

4. Reference no 5 should be written in English.

Response: Following your valuable feedback, in order to enhance the international scope of the article and facilitate retrieval and comprehension for international readers, we have replaced reference 5 with the corresponding English-language literature. We have ensured that the content of the cited English literature is consistent with the research results and conclusions of the original reference 5, to maintain the academic rigor and accuracy of the article.

5. In the material and method section, the reference for the migration rate formula in wound healing assay should be mentioned.

Response: Following your suggestion, we have added reference 13 to further support the migration rate formula we proposed in the wound healing assay. This reference provides methods for calculating the migration rate in wound healing assays, which aligns with our research methods, thereby enhancing the credibility and scientific rigor of our study results.

6. In figure 6 (A&B) and (F&G), it is preferred to put the statistical analysis of the western blot beside the blot figure in the same figure

Response: Thank you for your suggestion, we have integrated the results of the same panel onto a single figure for ease of review.
