# Supplementary material for: Inotodiol induces hepatocellular carcinoma apoptosis by activation of MAPK/ERK pathway
Source: PLoS One. 2025 Jan 29;20(1):e0318450. doi: 10.1371/journal.pone.0318450 (PMC11778785; doi:10.1371/journal.pone.0318450)
Supplement: Attachment — Submitted filename: Response to Reviewers.docx [file pone.0318450.s017.docx]

Jan 9th, 2025

Dear Dr. Wagdy M. Eldehna,

Thank you very much for your e-mail dated 12-Dec-2024.

Now I am sending our revised manuscript entitled “Inotodiol induces hepatocellular carcinoma apoptosis by activation of MAPK/ERK pathway” (Manuscript number: PONE-D-24-40336R1) for which we received your e-mail and reviewers’ comments 12-Dec-2024.

We have carefully revised our manuscript according the reviewer's comments. Please find a revised manuscript marked in **blue** showing major changes and the response to the reviewer's comments.

We hope this revised manuscript is acceptable.

With best regards,

Yours Sincerely

Yushuang Xing，

*Heilongjiang University of Traditional Chinese Medicine*

*Harbin 150040, China.*

*e-mail: 342937606@qq.com*

=====================================

**The responses to the Editor's comments:**

Journal Requirements:

Please review your reference list to ensure that it is complete and correct. If you have cited papers that have been retracted, please include the rationale for doing so in the manuscript text, or remove these references and replace them with relevant current references. Any changes to the reference list should be mentioned in the rebuttal letter that accompanies your revised manuscript. If you need to cite a retracted article, indicate the article’s retracted status in the References list and also include a citation and full reference for the retraction notice.

Response: Thank you for your feedback and suggestions. In response to the journal's requirements, we have conducted a thorough review of all the references cited in our manuscript. We have replaced two references and have also added the DOI numbers for four articles. The revised sections are indicated in blue within the revised manuscript for easy identification.

Additional Editor Comments:

The resolution of the figures is very low, in addition, the organization of the figures and the paper is still poor. Please address these issues.

Response: Thank you for your guidance. As requested, we have made the necessary adjustments to the figures in our manuscript. We have reorganized some of the images and increased their resolution to ensure that they meet the journal's standards for clarity and quality.

Richard Ente Claros:

Please provide the unadjusted and uncropped images underlying all blot and gel figures at this time.

Response: Thank you for your reminder. We have already uploaded the unadjusted and uncropped images to the Supporting Information and named them as required.

**The responses to the Reviewer's comments:**

Reviewer 1:
Comments to the Author

Thanks for the authors to address all the comments. The last thing I recommend is that authors should mention the toxic effect of this compound in the discussion since its actually toxic to the normal hepatic cells. Furthermore, the authors should also report if any of the animals showed signs of toxicity.

Response: Thank you for your valuable suggestions. We have added the effects of inotodiol on the body weight and organ mass of mice in the results section, and the results showed no significant differences. At the same time, we have expanded the discussion to include the toxicity of inotodiol's in vivo and in vitro effects, with the revised content highlighted in blue in the revised manuscript.
